# Development and content validation of a bodyweight-based HIIT training module for Chinese college students

**DOI:** 10.3389/fphys.2026.1797795

**Published:** 2026-06-10

**Authors:** Wendi Cui, Hao Li, Nor M. F. Farah, Arimi Fitri Mat Ludin

**Affiliations:** 1 Centre for Healthy Ageing & Wellness HCARE, Faculty of Health Sciences, Universiti Kebangsaan Malaysia, Kuala Lumpur, Malaysia; 2 Centre for Healthy Ageing & Wellness HCARE, Faculty of Health Sciences & Nan Hang Secondary School, Nanjing, China; 3 Centre for Community Health Studies ReaCH & Programme of Occupational Therapy, Faculty of Health Sciences, Universiti Kebangsaan Malaysia, Kuala Lumpur, Malaysia

**Keywords:** Chinese, college students, Delphi, HIIT, physical exercise

## Abstract

**Background:**

Physical fitness levels among Chinese college students have declined in recent years, representing a growing public health concern. High-intensity interval training (HIIT) is a time-efficient exercise modality with well-documented physiological benefits; however, validated HIIT training modules specifically tailored for Chinese college students remain limited.

**Purpose:**

This study aimed to develop and establish the content validity of a bodyweight-based HIIT training module tailored for Chinese college students.

**Methods:**

This methodological study employed a modified Delphi technique. HIIT movements were identified through a systematic literature review of studies published between 2014 and 2025. In the first round, 10 college physical education teachers screened the identified movements for feasibility and practicality. In the second round, nine experts evaluated the retained movements using Lawshe’s method to determine the Content Validity Ratio (CVR) and a four-point Likert scale to assess the Content Validity Index (CVI). Movements meeting the predefined validity thresholds were retained.

**Results:**

Twenty eligible studies were included, from which 48 HIIT movements were extracted. Following expert screening, 36 movements were excluded. Of the remaining 12 movements, eight (High Knees, Jumping Lunges, Jumping Jacks, Bodyweight Squats, Burpees, Butt Kicks, Mountain Climbers, and Push-ups) met the predefined criteria and were retained. All retained movements demonstrated acceptable item-level validity (I-CVI ≥ 0.78), and the overall scale-level validity was excellent (S-CVI = 0.976), indicating strong expert agreement.

**Conclusion:**

This study developed a bodyweight-based HIIT training module with excellent content validity for Chinese college students. The content-validated module provides a structured and accessible framework for exercise implementation and may serve as a foundation for future intervention studies to evaluate its effectiveness in improving physical fitness and related health outcomes.

## Graphical Abstract

**Figure:**
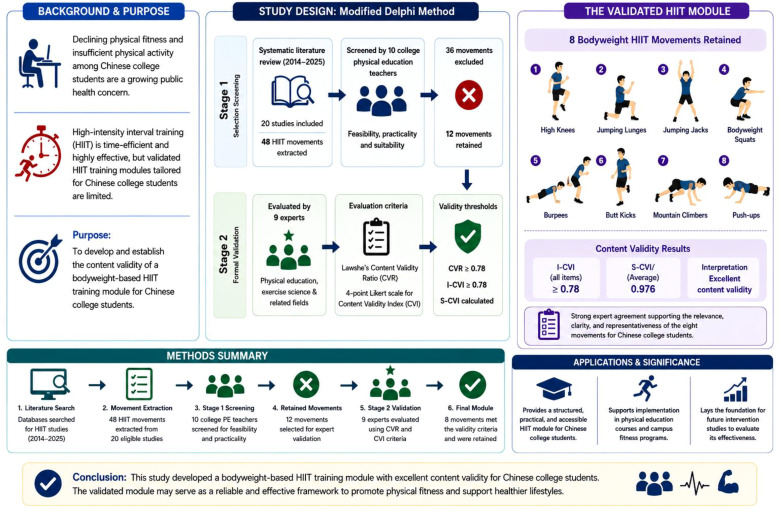

## 1 Introduction

Physical fitness is widely recognized as one of the most critical determinants of health across the lifespan (Jakicic, 2024). Adequate levels of cardiorespiratory fitness, muscular strength, muscular endurance, flexibility, and healthy body composition have been shown to effectively reduce the risk of chronic diseases, enhance immune function, and promote psychological well-being (Chiang et al., 2022; Bettariga et al., 2025). However, with rapid societal and technological development, declining physical fitness levels and insufficient physical activity have emerged as major global public health challenges (Bueno-Antequera and Munguía-Izquierdo, 2023).

At present, the physical fitness levels of Chinese college students have shown a continuous decline, characterized by reduced cardiorespiratory endurance, decreased muscular strength, and increasing prevalence of overweight and obesity (Dong et al., 2023). Multiple factors contribute to this trend, including increased academic demands, prolonged screen time, sedentary lifestyles, and limited opportunities for structured exercise (Huang et al., 2025). The college period is a critical window for the formation of lifelong health behaviors, and failure to establish positive habits during this stage may lead to persistent adverse health outcomes. Despite the widespread adoption of compulsory physical education courses in Chinese colleges, their impact on improving students’ physical fitness remains limited due to misaligned course objectives and insufficient engagement (Yang, 2025).

High-intensity interval training (HIIT) is an exercise modality characterized by short bouts of high-intensity activity interspersed with periods of active or passive recovery (Stavrinou et al., 2025). Substantial evidence indicates that HIIT can significantly improve cardiorespiratory fitness, muscular strength, metabolic health, and body composition (Scoubeau et al., 2022). In addition to physiological benefits, HIIT has been associated with improved exercise enjoyment and motivation, which are key determinants of long-term adherence (Ekkekakis and Biddle, 2023). Moreover, HIIT requires minimal time commitment, has few environmental constraints, and can be performed without specialized equipment, making it particularly suitable for implementation among college students within the college setting (Santoso and Boenyamin, 2019).

Although the literature supporting the effectiveness of HIIT has grown substantially, most previous studies have primarily focused on short-term intervention outcomes. In contrast, research on the systematic development of HIIT training modules remains limited. Notably, there is a lack of HIIT training modules specifically designed for and validated among Chinese college students. Therefore, the present study aimed to develop and validate a bodyweight-based HIIT training module tailored to this population. Future clinical trials are planned to evaluate the effectiveness of the proposed training module.

## 2 Methods

### 2.1 Study design

This study employed a methodological design using a modified Delphi technique to develop and establish the content validity of a bodyweight-based HIIT training module for Chinese college students. The study was conducted in two sequential phases: (1) identification of candidate HIIT movements through a systematic literature review, and (2) a modified Delphi technique consisting of a preliminary feasibility screening stage followed by a formal expert content-validation stage.

All participating experts and college physical education teachers voluntarily participated in the study and provided informed consent prior to participation. The study protocol was reviewed and approved by the Universiti Kebangsaan Malaysia Research Ethics Committee (Reference No. JEP-2025-206). No clinical intervention or patient related data were involved.

### 2.2 Phase 1: identifying HIIT movements

#### 2.2.1 Literature search

A systematic literature search was conducted to identify HIIT movements applicable to college students. Three electronic databases (Scopus, PubMed, and Web of Science) were searched for studies published between 2014 and 2025. The search strategy combined terms related to population (“college students” OR “university students”) and intervention (“high-intensity interval training,” “HIIT,” “high-intensity interval exercise,” and related synonyms). The full search strings are presented in **Table 1**. The search and selection process followed a structured screening procedure, including duplicate removal, title and abstract screening, and full-text review. A total of 532 records were initially identified. The database search was conducted between December 2025 and January 2026.

**Table 1:** List of search string.

Database	Search String
Scopus, PubMed, Web of Science	(“college students” OR “university students”) AND (“high-intensity interval training” OR “HIIT” OR “high-intensity interval exercise”)

#### 2.2.2 Literature inclusion and exclusion criteria

Studies were eligible for inclusion if they: (1) were original research articles published in English, (2) included college students as participants, and (3) implemented bodyweight-based HIIT interventions. Studies were excluded if they: (1) were not published in English, (2) involved populations other than college students, or (3) did not involve bodyweight-based HIIT interventions.

#### 2.2.3 Literature screening and quality assessment

**Figure 1:**
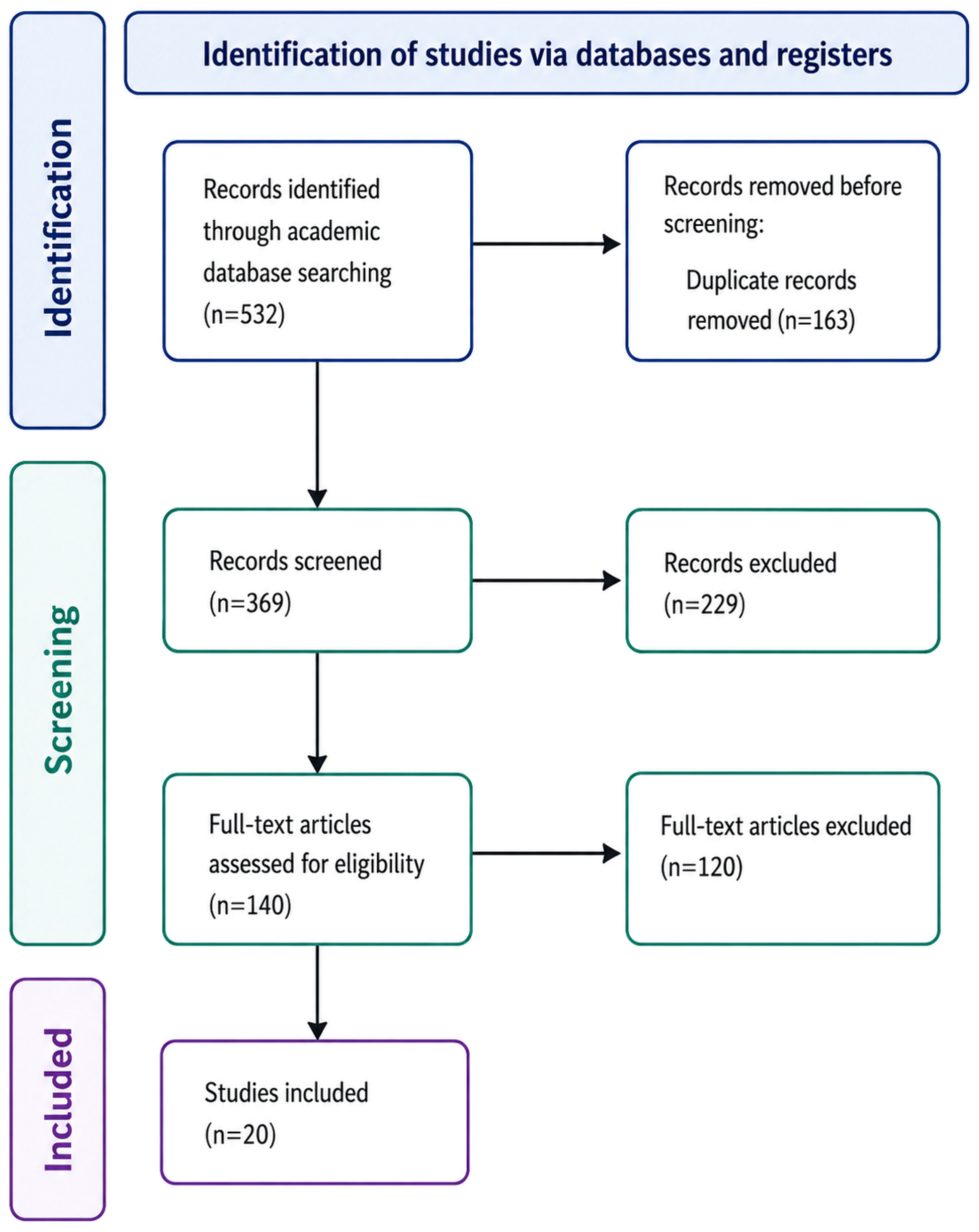
PRISMA flow diagram.

After removing duplicate articles, 369 records remained and were screened based on titles and abstracts. Following this process, 140 articles were considered potentially eligible and were subjected to full-text review. Ultimately, 20 studies met the predefined inclusion criteria and were included in the final analysis. The process of screening and selecting the relevant articles is reported in the PRISMA flow chart (**Figure 1**).

All included studies were independently assessed for methodological quality by two researchers with expertise in evidence-based nursing. In cases of disagreement, a third evidence-based nursing researcher was consulted to resolve discrepancies. All included studies were published within the past decade and comprised 19 randomized controlled trials (RCTs) and one cross-sectional study. From these studies, 48 distinct HIIT movements were extracted, including 28 standing movements and 20 prone-based movements. Detailed information is presented in **Table 2**.

**Table 2:** Summary of systematic review articles.

No	Study	HIIT movement		Frequency	Intensity	Time/duration
		Standing movement	Prone type movement			
1	(Tanucan Miller et al., 2023)	High Knees, Skaters, Tuck Jumps, Squat Jump, Burpees	Sprawl, Knee Tuck to Pushup, Mountain Climbers	N/R	N/R	10-weeks
2	(Dregney et al., 2023)	Jump squats, high knees, jumping lunges, burpees	Plank variations	N/R	70–85% maximum heart rate	N/R
3	(Chang et al., 2024)	Shuttle run		3 times/week	90% of Maximal Aerobic Speed	3 months
4	(Masagca, 2024)	Jumping Jаcks, BW Squаts, Jumping Squаts, Burpееs, Mаximаl Jump, Lungеs, High knееs, Cаlf jump, Butt kicks, Jump Lungеs, Sidе plаnk R, Sidе plаnk L, Lаtеrаl Jumps, Stаtic Lungе, hingе аnd squаt, Fоrwаrd Lungе	Push ups, Mоuntаin climbеr, Russiаn Twist, Bicyclе crunchеs, Plаnk shоuldеr tаps, Fluttеr kicks, Dеаd bug pоsitiоn, Glutе bridgе, Lеg Rаisе	N/R	N/R	10-weeks
5	(Biswal and Anandhi, 2024)	High knees, Butt kicks, jumping jacks, Chest expansion, raised arm circles, raised leg swing Knee extension	Alternate arm/leg raise Side leg raise	3 times/week	N/R	8-weeks
6	(Wang et al., 2024)	Squat, jumping jacks, high knees, burpees		N/R	N/R	N/R
7	(Lu et al., 2023)	Jumping jacks, high knees, squat jumps	Mountain climbers	3 times/week	HRmean above the 80%	12-weeks
8	(Philippot et al., 2022)	jump squats, burpees, jump forward lunge, jumping jack	mountain climbers	3 times/week	HRmax at least 80%	4-weeks
9	(Sun et al., 2025)	*in situ* deep squat, cross quadrant jump, step open and close, *in situ* high leg raise, square contact run, alternating squatting on both the left and right sides, alternating lunge squatting with support leg lifted, semi-squat jump up		2 times/week	Heart rate ranging from 130 to 150	8-weeks
10	(Lu et al., 2021)	Jumping Jacks, High knees, Side to side squat, Burpees, Deep squat jumps, Butt kickers	Mountain climbers, Forearm plank to high plank	3 times/week	HRmax at least 75%	12-weeks
11	(Lu et al., 2022)	jumping jacks, high knees, squat jumps	mountain climbers	3 times/week	N/R	2-weeks
12	(Dregney et al., 2025)	jump squats, high knees, jumping jacks	plank variations, push-ups	3 times/week	N/R	8-weeks
13	(Kaur and Rizvi, 2024)	squats, deadlifts	plank, push-ups	5 times/week	N/R	6-weeks
14	(Hu et al., 2022)	jumping jacks, run-ups, squat jump, burpee, step jump, double knee lifts, lunges in place	high elbow plank, mountain climbers	5 times/week	90% of the maximal heart rate	4-weeks
15	(Fu et al., 2025)	opening and closing jumps, squats, step jumps, poppy jumps, lunge squats	push-ups, supine and bent-knee raises, hip bridges	5 times/week	over 90% VO_2_max	4-weeks
16	(Rhibi et al., 2022)	Running and recovery intervals		3 times/week	100% maximal aerobic velocity (MAV) and 110% MAV	8-weeks
17	(Zuo et al., 2025)	jumping jacks, kickbacks, underarm claps, squat jumps, high knees, sidekicks	mountain climbers, knee drops	3 times/week	85%- 95% of HRmax	8-weeks
18	(Sukri et al., 2024)	jumping jack, lunges, squat, standing elbow to knee crunch	sit up	3 times/week	N/R	12-weeks
19	(Miller et al., 2023)	High Knees, Skaters, Tuck Jumps, Squat Jump, Burpees	Sprawl, Knee Tuck to Pushup, Mountain Climbers	N/R	N/R	10-weeks
20	(Boer, 2019)	Circular running		3 times/week	90% of HRmax	7-weeks

### 2.3 Phase 2: selection and validation of HIIT movements

This study adopted a modified Delphi approach consisting of two sequential stages. The first stage served as a preliminary expert screening process, while the second stage constituted the formal Delphi validation.

In the initial stage, 10 college physical education instructors participated in face-to-face discussions to evaluate the feasibility, practicality, and appropriateness of the identified HIIT movements. This step aimed to refine the initial pool of movements by removing those considered unsuitable for the target population.

In the second stage, a panel of nine experts was invited to participate in the formal Delphi process. Experts independently evaluated each retained movement using structured rating criteria. Anonymity was maintained during this stage to minimize bias and ensure independent judgment (Humphrey-Murto et al., 2020).

The results from the initial screening stage were systematically incorporated into the Delphi process by retaining only those movements deemed feasible for further evaluation. The use of different participant groups reflects their distinct roles, with practitioners contributing to feasibility assessment and experts providing formal content validation.

#### 2.3.1 Expert selection criteria

All invited experts were in-service college physical education instructors with valid higher education teaching qualifications. Each expert had more than five years of teaching experience at the tertiary level. Their extensive professional experience enabled them to develop a comprehensive understanding of college students’ physical activity habits and characteristics, thereby ensuring that the module was appropriately tailored to the needs of the target population (Reeve and Shin, 2020).

#### 2.3.2 Implementation of Delphi method

Expert feedback was collected using the Delphi method. Previous methodological studies have suggested that a panel of 8–20 experts is sufficient to achieve reliable consensus in Delphi research (Hsu and Sandford, 2007; Skulmoski et al., 2007). Given the specific focus of this study on HIIT exercise selection and the high level of expertise and homogeneity among the panel members, 9 experts were considered adequate for content validity evaluation.

In the first round, 10 college physical education teachers conducted face-to-face evaluations to determine whether each movement should be included or excluded. In the second round, nine experts participated in the formal validation process. To minimize potential bias, the first-stage face-to-face discussion was limited to preliminary feasibility assessment and did not involve formal content validity scoring. During the second-stage Delphi validation, experts independently completed anonymous online evaluations without direct interaction with other panel members. Feedback from the preliminary stage was summarized and incorporated into the second-round evaluation process. Consensus was predefined using quantitative thresholds of CVR ≥ 0.78 and I-CVI ≥ 0.78.

#### 2.3.3 Validation procedure

Following Phase 1’s preliminary screening, the selected HIIT movements were uploaded to the wjx.cn platform to form a structured questionnaire. A panel of nine specialists in physical education, sports training, and social sports guidance independently assessed each movement according to Lawshe’s method, categorizing them as ‘essential,’ ‘useful but not essential,’ or ‘not necessary.’ Finally, the ratings were logged in Microsoft Excel to compute each item’s Content Validity Ratio (CVR).”

The Content Validity Index (CVI) was subsequently used to assess the relevance and representativeness of the retained movements. Experts rated each movement using a four-point Likert scale ranging from 1 (not relevant) to 4 (highly relevant). These ratings were also recorded in Microsoft Excel to calculate item level and scale-level CVI values.

#### 2.3.4 Statistical methods

Content validity was assessed using both the Content Validity Ratio (CVR) and Content Validity Index (CVI). CVR was calculated based on Lawshe’s method to determine whether each movement was essential, using the formula: CVR = (n_e_ − N/2)/(N/2), where n_e_ is the number of experts indicating “essential” and N is the total number of experts. Based on Lawshe’s critical values for nine experts, a minimum CVR of 0.78 was required for item retention (Ayre and Scally, 2014).

The Content Validity Index (CVI) was calculated at both the item level (I-CVI) and scale level (S-CVI). I-CVI was defined as the proportion of experts rating an item as either “relevant” or “highly relevant” (ratings of 3 or 4 on a 4-point Likert scale). An I-CVI threshold of ≥0.78 was considered acceptable. The S-CVI value was calculated based exclusively on the eight retained exercises included in the final HIIT module, with a value ≥0.90 indicating excellent content validity. These criteria were based on established methodological recommendations (Polit and Beck, 2006).

This combination of Delphi consensus and quantitative content validity indices represents a well-established approach for the systematic development and validation of training modules and intervention components.

## 3 Results

### 3.1 General information of experts

The nine experts included in round 2 were aged between 33 and 45 years and had 7–15 years of professional experience (10.56 ± 2.79 years). Among them, two held doctoral degrees and seven held master’s degrees. Regarding academic specialization, four experts specialized in sports training, three in physical education, and two in social sports guidance. The socio-demographic characteristics of the experts are presented in **Table 3**.

**Table 3:** Socio-demographic details of experts.

Variables	N
**Working experience in the field [Mean (SD) in years]**	**10.56 (2.79)**
Gender	
Male	**6**
Female	**3**
Professional specialization	
Sports training	**4**
Physical education	**3**
Social sports guidance	**2**

Bold values indicate the number of experts.

### 3.2 Degree of harmonization of expert opinions

During the first round of expert evaluation, 36 movements were excluded because of concerns related to execution difficulty and limited practical applicability. In the second round, experts evaluated the remaining 12 HIIT movements, with the results presented in **Table 4**. Ultimately, eight movements (High Knees, Jumping lunges, Jumping Jacks, Bodyweight Squats, Burpees, Butt kicks, Mountain Climbers, Push-ups) were retained for inclusion in the final HIIT training module. All I-CVI values exceeded 0.78, and the S-CVI was 0.976, which is higher than the recommended threshold of 0.90, detailed information is presented in **Table 5**. These findings provide preliminary evidence that the training module is content validated from an expert perspective and may be practically applicable, although further feasibility testing is needed to confirm its implementation in practice. Although several removed exercises demonstrated acceptable I-CVI values, they failed to achieve the predefined CVR threshold required for retention. This indicates that experts considered these exercises relevant but not sufficiently essential for inclusion in the final module.

**Table 4:** Elements of HIIT training module.

Elements of HIIT training module	Expert evaluation			CVR	Remark
	Essential	Useful but not essential	Not necessary		
High knees	9	–	–	1	Retained
Squat Jumps	5	4	–	0.11	Removed
Jumping lunges	8	1	–	0.78	Retained
Jumping Jacks	9	–	–	1	Retained
Bodyweight Squats	9	–	–	1	Retained
Burpees	9	–	–	1	Retained
Calf jump	7	2	–	0.56	Removed
Butt kicks	9	–	–	1	Retained
Side to side squat	6	3	–	0.33	Removed
Sidekicks	3	6	–	-0.33	Removed
Mountain Climbers	9	–	–	1	Retained
Push ups	9	–	–	1	Retained

**Table 5:** CVI of HIIT training module.

Elements of HIIT training module	Expert evaluation				I-CVI
	Not relevant	Somewhat not relevant	Relevant	Highly relevant	
High knees	–	–	2	7	1
Squat Jumps	–	–	5	4	1
Jumping lunges	–	–	2	7	1
Jumping Jacks	–	–	5	4	1
Bodyweight Squats	–	–	1	8	1
Burpees	–	–	3	6	1
Calf jump	–	1	3	5	0.89
Butt kicks	–	–	4	5	1
Side to side squat	–	–	6	3	1
Sidekicks	–	1	4	4	0.89
Mountain Climbers	–	–	1	8	1
Push ups	–	–	2	7	1
S-CVI					0.976

During the first-stage screening, movements were primarily excluded due to concerns related to technical complexity, safety, and practicality for the target population. Exercises requiring advanced coordination, high skill levels, or specialized supervision were considered less suitable for general college students. Additionally, movements associated with higher injury risk, particularly those involving excessive joint loading or unstable movement patterns, were removed. Experts emphasized the importance of selecting exercises that are easy to learn, safe to perform in unsupervised or group settings, and adaptable to varying fitness levels.

Although a high level of agreement was observed for most retained movements, minor variations in expert ratings were noted during the validation process. These differences were primarily related to perceived intensity and suitability for beginners. However, consensus was achieved through quantitative thresholds defined by CVR and CVI criteria, ensuring that only movements meeting established validity standards were retained.

### 3.3 Structure of the HIIT training module

**Figure 2:**
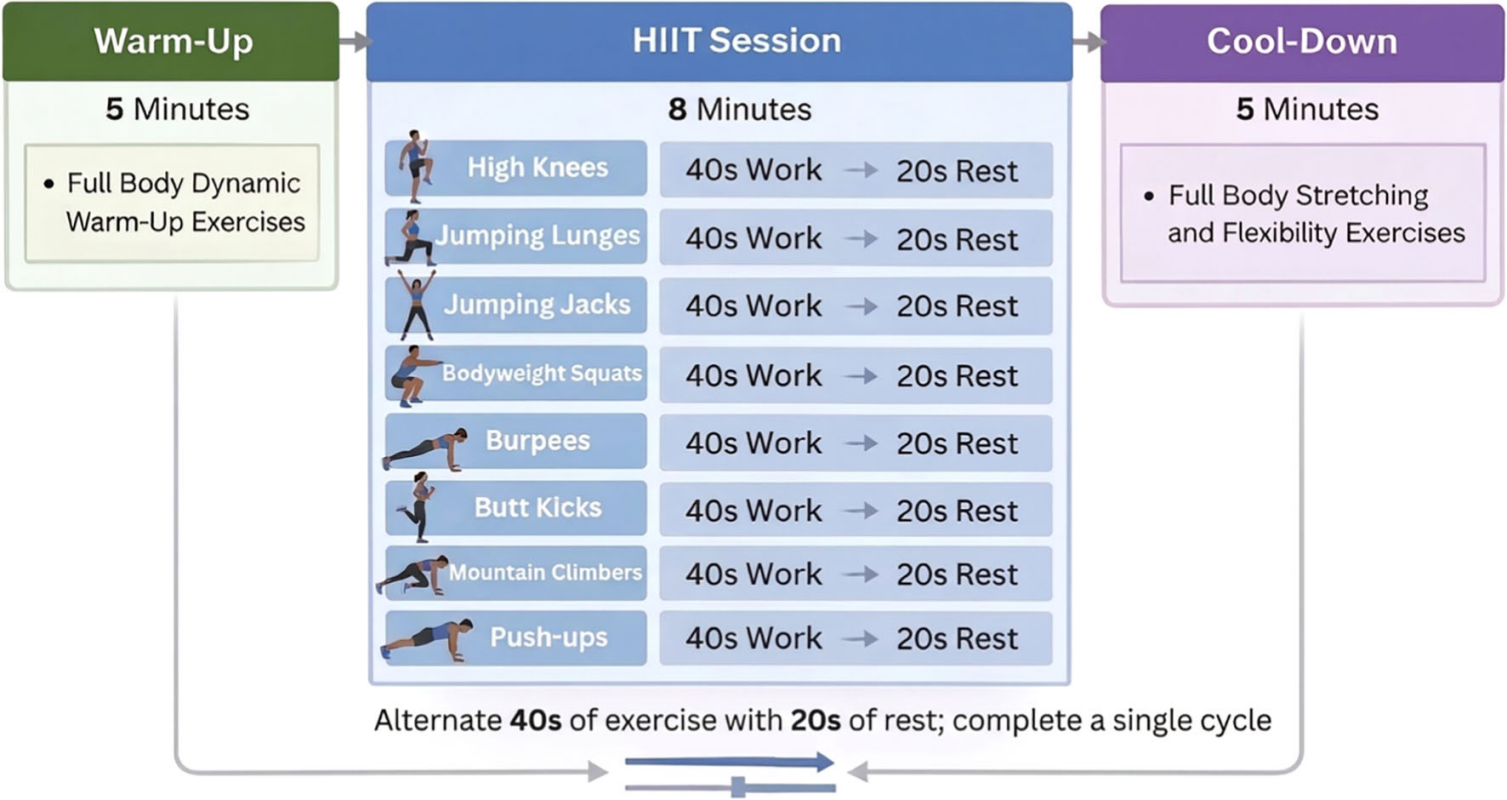
Structure of the validated bodyweight-based HIIT training module.

The final HIIT training module consists of eight bodyweight exercises arranged in a structured format comprising a warm-up, high-intensity interval training phase, and cool-down. The module is planned to be performed three times per week, with each session lasting 18 minutes. Each session includes a 5-minute warm-up, 8 minutes of HIIT training, and a 5-minute cool-down. During the HIIT phase, participants are expected to reach an intensity of 75–90% of maximal heart rate (HRmax). The exercises are designed to be performed sequentially with minimal equipment, allowing flexible implementation in various settings. The overall structure of the training module is illustrated in **Figure 2**. Training progression will be achieved by gradually increasing the number of repetitions.

The final set of eight exercises represents a balanced combination of fundamental movement patterns and key components of health-related physical fitness. Specifically, exercises such as High Knees and Jumping Jacks primarily target cardiorespiratory endurance, while Bodyweight Squats and Jumping Lunges contribute to lower-body muscular strength and endurance. Push-ups and Mountain Climbers engage upper-body and core musculature, whereas Burpees and Butt Kicks provide full-body dynamic engagement. This combination ensures that the HIIT module addresses multiple dimensions of physical fitness within a time-efficient format.

Overall, the selection process was guided by a combination of empirical evidence from the literature and practical considerations derived from expert experience, resulting in a module that is both scientifically grounded and applicable to real-world college settings.

## 4 Discussion

This study systematically developed and validated a HIIT training module specifically designed for Chinese college students using a Delphi-based expert consensus approach. The study aimed to address the growing public health concern regarding declining physical fitness levels among Chinese college students. The main findings indicate that, following a comprehensive literature review and a two-phase expert evaluation process, eight HIIT movements were retained, and the final module demonstrated excellent content validity, as reflected by high I-CVI values (≥0.78) and an S-CVI of 0.976. These findings support the scientific rigor and practical relevance of the proposed HIIT training module.

College students represent an important target group for physical activity promotion because health behaviors established during this stage often continue into adulthood (Scroggs et al., 2025). The validated HIIT module addresses this need by providing a time-efficient and accessible training framework that can be implemented within existing college infrastructures, such as compulsory physical education courses and campus-wide health promotion programs.

The substantial reduction in the number of movements during the expert screening phase reflects an emphasis on feasibility, practicality and safety. The excluded movements were considered too difficult for general Chinese college students or impractical for them to perform. When exercise tasks are overly difficult to perform, college students may experience reduced interest in physical activity and develop psychological barriers, which in turn lead to lower overall participation level (Alkhawaldeh et al., 2024). In contrast, the retained movements predominantly involve fundamental movement patterns (e.g., squatting, pushing, jumping, and locomotion) that are easily taught, require minimal supervision, and are suitable for individuals with varying fitness levels. The high degree of expert consensus observed in the content validity assessment underscores the relevance of the selected movements for Chinese college students. And the use of both CVR and CVI metrics allowed for a nuanced evaluation of exercise necessity and relevance, strengthening confidence in the final composition of the module.

HIIT has been shown to provide a wide range of benefits across diverse populations. In young adults and college students, HIIT is associated with improvements in cardiorespiratory fitness, body composition, and psychological well-being (Lu et al., 2021; Chang et al., 2024). Among older adults, appropriately modified HIIT programs can enhance functional capacity, muscular strength, and cardiovascular health, supporting healthy aging (Marriott et al., 2021). In clinical and at-risk populations, including individuals with obesity or metabolic disorders, HIIT has been linked to improvements in glucose regulation, insulin sensitivity, and overall metabolic health (Gallo-Villegas et al., 2022).

Although HIIT has been widely shown to improve multiple health-related outcomes in college-aged populations, most prior studies have focused on intervention efficacy rather than the validation of training content (Yin et al., 2025). There is substantial heterogeneity and limited transparency in training protocols. For instance, in the study by Tanucan, Miller et al. (2023), the intervention was described as a Tabata protocol but lacked detailed reporting of exercise selection criteria and progression strategy. Similarly, Chang, Abdul Malik et al. (2024) compared moderate and high-intensity interval exercise but provided limited justification for the specific movement selection. The present study complements this body of evidence by shifting attention to the foundational stage of intervention development. In contrast to these efficacy-focused studies, the present study adopts a development-oriented approach by systematically identifying, screening, and validating HIIT movements using a combination of evidence synthesis and expert consensus. The final module consisted exclusively of bodyweight exercises that required no equipment. For college students, this approach offers several advantages, including the ability to exercise anytime and anywhere without the need for financial investment or time spent traveling to a gym. In addition, these exercises do not require large or specialized training spaces. Multiple studies have demonstrated that limitations related to exercise facilities, equipment availability, time constraints, and financial conditions significantly influence levels of physical activity participation (Pedersen et al., 2021; Griffiths et al., 2022). Consequently, the proposed training module represents a cost-effective, time-efficient, and accessible exercise modality for college students.

The strengths of this study include the systematic integration of literature evidence with expert consensus, clearly defined expert selection criteria, and the application of established content validity indices. However, several limitations should be considered when interpreting the findings of this study. First, the present study focused exclusively on content validity and expert consensus; therefore, no conclusions can be drawn regarding the effectiveness of the HIIT training module in improving physical fitness or related health outcomes.

Second, no pilot testing was conducted among the target population. As such, feasibility, usability, adherence, and user experience—including enjoyment and perceived exertion—were not evaluated. Third, the reliability and repeatability of the module were not assessed, and therefore consistency of implementation across different settings remains unknown.

Additionally, safety outcomes and potential injury risks were not empirically examined. Although expert evaluation considered feasibility and appropriateness, real-world application may reveal further considerations. The current module also does not account for potential differences based on sex, baseline fitness level, or prior exercise experience, which may influence exercise tolerance and adaptation.

Furthermore, the expert panel was relatively homogeneous in professional background, which may have limited the diversity of perspectives during the validation process. The selected exercises, while designed to be broadly applicable, may not fully capture the heterogeneity of Chinese college students in terms of physical ability, fitness level, and training experience.

These limitations are consistent with the developmental nature of the present study. Ongoing and future research will address these gaps through feasibility and pilot studies, followed by randomized controlled trials to evaluate the effectiveness, safety, adherence, and practical implementation of the validated HIIT training module.

### 4.1 Future directions

The present study was intentionally designed as a standalone methodological investigation focusing on the development and content validation of the HIIT training module. This approach aligns with best practices in intervention research, where the development and validation phase is conducted prior to efficacy testing. By establishing content validity through a systematic and expert-informed process, the current study provides a robust foundation for subsequent experimental studies. Ongoing and future research will evaluate the effectiveness, feasibility, and adherence of the proposed module through well-designed intervention trials. Combining intervention development and effectiveness evaluation within a single manuscript may compromise methodological clarity. According to established frameworks for complex intervention research, such as the Medical Research Council (MRC) guidance, intervention development and evaluation are recommended to be conducted and reported as distinct phases to ensure transparency and rigor (Craig et al., 2008; Skivington et al., 2021).

## 5 Conclusion

This study developed and established the content validity of a bodyweight-based HIIT training module for Chinese college students using a systematic and expert-informed approach. From an initial pool of 48 identified movements, eight exercises (High Knees, Jumping Lunges, Jumping Jacks, Bodyweight Squats, Burpees, Butt Kicks, Mountain Climbers, and Push-ups) were retained following modified Delphi technique.

All retained movements demonstrated acceptable item-level content validity (I-CVI ≥ 0.78), and the overall scale-level content validity was excellent (S-CVI = 0.976), indicating a high level of expert consensus.

These findings provide preliminary support for the relevance and potential applicability of the proposed module as a structured exercise framework for Chinese college students. The content-validated module may serve as a useful basis for future intervention studies examining its feasibility, effectiveness, and impact on physical fitness and related health outcomes.

## Data Availability

The original contributions presented in the study are included in the article/supplementary material. Further inquiries can be directed to the corresponding authors.
